# Short-Term Supplementation of Dietary Arginine and Citrulline Modulates Gilthead Seabream (*Sparus aurata*) Immune Status

**DOI:** 10.3389/fimmu.2020.01544

**Published:** 2020-08-05

**Authors:** Lourenço Ramos-Pinto, Rita Azeredo, Carlota Silva, Luís E. C. Conceição, Jorge Dias, Daniel Montero, Silvia Torrecillas, Tomé S. Silva, Benjamin Costas

**Affiliations:** ^1^Centro Interdisciplinar de Investigação Marinha e Ambiental (CIIMAR), Universidade Do Porto, Terminal de Cruzeiros Do Porto de Leixões, Matosinhos, Portugal; ^2^Instituto de Ciências Biomédicas Abel Salazar (ICBAS-UP), Universidade Do Porto, Porto, Portugal; ^3^SPAROS Lda., Área Empresarial de Marim, Olhão, Portugal; ^4^Grupo de Investigación en Acuicultura (GIA), IU-ECOAQUA, Universidad de Las Palmas de Gran Canaria, Las Palmas, Spain

**Keywords:** amino acids, immunology, sustainable aquaculture, functional feeds, plasma proteome

## Abstract

Several amino acids (AA) are known to regulate key metabolic pathways that are crucial for immune responses. In particular, arginine (ARG) appears to have important roles regarding immune modulation since it is required for macrophage responses and lymphocyte development. Moreover, citrulline (CIT) is a precursor of arginine, and it was reported as an alternative to ARG for improving macrophage function in mammals. The present study aimed to explore the effects of dietary ARG and CIT supplementation on the gilthead seabream (*Sparus aurata*) immune status. Triplicate groups of fish (23.1 ± 0.4 g) were either fed a control diet (CTRL) with a balanced AA profile, or the CTRL diet supplemented with graded levels of ARG or CIT (i.e., 0.5 and 1% of feed; ARG1, CIT1, ARG2, and CIT2, respectively). After 2 and 4 weeks of feeding, fish were euthanized and blood was collected for blood smears, plasma for humoral immune parameters and shotgun proteomics, and head-kidney tissue for the measurement of health-related transcripts. A total of 94 proteins were identified in the plasma of all treatments. Among them, components of the complement system, apolipoproteins, as well as some glycoproteins were found to be highly abundant. After performing a PLS of the expressed proteins, differences between the two sampling points were observed. In this regard, component 1 (61%) was correlated with the effect of sampling time, whereas component 2 (18%) seemed associated to individual variability within diet. Gilthead seabream fed ARG2 and CIT2 at 4 weeks were more distant than fish fed all dietary treatments at 2 weeks and fish fed the CTRL diet at 4 weeks. Therefore, data suggest that the modulatory effects of AA supplementation at the proteome level were more effective after 4 weeks of feeding and at the higher inclusion level (i.e., 1% of feed). The bactericidal activity increased in fish fed the highest supplementation level of both AAs after 4 weeks. Peripheral monocyte numbers correlated positively with nitric oxide, which showed an increasing trend in a dose-dependent manner. The colony-stimulating factor 1 receptor tended to be up-regulated at the final sampling point regardless of dietary treatments. Data from this study point to an immunostimulatory effect of dietary ARG or CIT supplementation after 4 weeks of feeding in the gilthead seabream, particularly when supplemented at a 1% inclusion level.

## Introduction

Feeds can have significant health implication in farmed fish and thus good practices in diet formulation are imperative, since it also represents a significant expenditure to the aquaculture industry. Therefore, in recent years, the industry is willing to explore the so called “functional feeds,” fortified diet formulations that have added benefits besides meeting fish essential nutritional requirements, being both health and growth boosters (1, 2). The application of such diets can provide a healthier and more sustainable alternative to chemotherapeutic and antibiotic treatments. Amongst a wide range of candidate functional ingredients, little attention has been paid to the role of individual amino acids as potential immunomodulators in fish. In this regard, arginine (ARG) is one of the most versatile amino acids by serving as the precursor for the synthesis of protein, nitric oxide (NO), urea, polyamines, proline, glutamate, creatine and agmatine in terrestrial animals (3). Polyamines are important for lymphocytes proliferation and differentiation, and NO is a strong bactericidal agent synthetized by activated phagocytes.

Considerable evidence from studies in diverse animal models indicates that adequate amounts of arginine are required for lymphocyte development and that a dietary arginine surplus enhances immune function during immunological challenges (4). Nevertheless, arginine was also reported to mediate immunosuppressive mechanisms. In mammals, T-cell activation and function is dictated by arginine metabolism in myeloid suppressor cells (5). Sharma et al. (6) observed that L-arginine, by its conversion to NO, was able to modulate the immune response in rats and mice under restraint stress (RS), antagonizing the immunosuppressive effect of RS on humoral as well as cell-mediated immune responses. In a similar way to higher vertebrates, fish produce NO and ornithine from arginine *via* the inducible NO synthase (iNOS) and arginase, respectively (7, 8). Indeed, upon inflammatory circumstances, fish phagocytes produce NO, acting as an oxidant against pathogens compromising its structures and function (5, 9). In fish, different outcomes have been observed. A positive effect of feeds supplemented with arginine on disease resistance has been reported in several teleosts (2, 10), whereas an inhibitory effect was observed in Jian carp (*Cyprinus carpio var. Jian*) where both *in vivo* and *in vitro* arginine supplementation counteracted LPS-induced inflammatory responses (11). A similar detrimental effect was also observed in European seabass (*Dicentrarchus labrax*) fed arginine-enriched diets (12). New insights of a recent study revealed that arginine supplementation could compromise to some extent the seabass cell-mediated immune response, decreasing the circulating numbers of neutrophils and monocytes (13). Therefore, despite current knowledge about arginine metabolism, opposing effects from different studies point to a species-specific role of arginine in the fish immune status, a topic that deserves further attention.

Arginine and its metabolites (L-ornithine and L-proline) have also been suggested to play a relevant role in the stress response, promoting stress mitigation in different animals. In fact, dietary arginine supplementation decreased the serum level of cortisol (around−33% than the CTRL fed group) in growing-finishing pigs and weaned piglets (14, 15). Experiments with Senegalese sole (*Solea senegalensis*) under chronic stressful conditions (e.g., high densities and handling), reported that stress can affect amino acids requirements (16) Arginine and histidine concentration were significantly lower in fish under crowding stress (17). This confirms that during stressful conditions, essential amino acids involved in metabolic pathways have an important role. In this regard, ARG-rich diets proved to decrease plasma cortisol levels and enhance several aspects of the innate immune response (i.e., circulating monocytes, NO production and humoral parameters) in Senegalese sole under chronic stress, increased disease resistance upon a possible bacterial infection (10, 18).

Citrulline (CIT) is the precursor of arginine, a two-steps reaction mediated by argininosuccinate synthase and argininosuccinate lyase. It is also a by-product of arginine upon conversion to NO (19), a mechanism also described in fish (20). In mammals, it has been reported that citrulline supplementation might offer a safe alternative to arginine for improving macrophage function under certain metabolic conditions. Moreover, Batista et al. (21) found that a citrulline-enriched diet improved gut function by decreasing intestinal permeability and decreasing bacterial translocation improving protection against bacterial, and immune function in mice; and it stimulated intestinal production of secretory immunoglobulin A, which is the first line of host defenses against environmental pathogens.

Indeed, few studies have approached the effect of citrulline surplus on immune responses in higher vertebrates, and to the best of our knowledge there is scarce information about this in fish. Nonetheless, Buentello and Gatlin (7) revealed that *in vitro* NO production was improved in peritoneal macrophages of channel catfish, *Ictalurus punctatus*, upon addition of citrulline to the culture media. A pathogen challenge performed by the same authors revealed that dietary arginine surplus (2% inclusion of the diet) enhanced the ability of channel catfish to survive after exposure to *Edwardsiella ictaluri* (22).

Presently, the potential use of these amino acids as dietary supplements in fish health management is not fully developed. Citrulline could present itself as an additive to improve macrophage-mediated immune responses, but few studies have been performed in fish so far. Therefore, the potential immunomodulatory role of arginine and citrulline in fish deserves further attention, and efforts should be driven to ascertain local and systemic immune responses and disease resistance for each particular species of interest. The present study aimed to explore the response of gilthead seabream (*Sparus aurata*) juveniles to arginine- and citrulline-enriched diets in terms of their health status, in the context of a practical feed formulation.

## Materials and Methods

### Rearing Conditions

The feeding trial was carried out at the experimental facilities of the IU-ECOAQUA of the Universidad de Las Palmas de Gran Canaria (Las Palmas, Canary Islands, Spain). Gilthead seabream juveniles (*Sparus aurata*), originated from the natural spawning of wild broodstock were reared according to standard larval and juvenile rearing protocols at the experimental facilities of GIA (Grupo de Investigación en Acuicultura) until achieving the desired size (IU-ECOAQUA).

Fish with an initial body weight of 23.08 ± 0.33 g (mean ± SD) were randomly distributed in 500 L tanks and fed a commercial diet for 3 weeks to ensure acclimation to the experimental conditions. Triplicate groups of 80 gilthead seabream per treatment were hand-fed *ad libitum* three times a day (except Sundays, when fish were fed once a day) each experimental diet for 1 month. The trial was carried out in a RAS system with aerated seawater (temperature: 22 ± 0.5°C, salinity of 37 ± 1‰; pH of 8.2 ± 0.2; photoperiod: 12L/12D). Water flow was 15–20 L/min, oxygen content in water effluents was always higher than 90% saturation and unionized ammonia was regularly recorded and remained below toxic levels (<0.05 mg/L). All physical and chemical water parameters were evaluated daily during the experiment.

The animal experiments described complied with the guidelines of the European Union Council (2010/63/EU) for the use of experimental animals.

### Diets Formulation

Extruded feeds were based on plant proteins with 5% of fish meal inclusion and a partial replacement (30–35%) of fish oil. This diet formulation mimics most of the currently used commercial diets for gilthead seabream. Based on this formulation, 5 experimental diets with varying concentrations of arginine and citrulline were produced at SPAROS Lda. (Olhão, Portugal).

A control (CTRL) diet was formulated similar to commercial diet for this species. The four other diets were identical to the CTRL diet but supplemented with graded levels of arginine and citrulline at 0.5% (ARG1 and CIT1) and 1% (ARG2 and CIT2) of feed (Tables 1 and 2). Main ingredients were ground (below 250 μm) in a Hosakawa, model #1 micropulverizer hammer mill (Hosokawa Micron Ltd., United Kingdom). These ground ingredients were then mixed according to the target formulation in a Double-helix Mixture TGC, model 500 L (TGC Extrusion, France), to attain a basal mixture (no oils were added at this stage). All diets were manufactured by extrusion (pellet size 2.0 mm) by means of a pilot-scale twin-screw extruder CLEXTRAL BC45 (Clextral, France) with a screw diameter of 55.5 mm and temperature ranging 105–110°C. Upon extrusion, all batches of extruded feeds were dried in a convection oven (OP 750-EF, LTE Scientifics, United Kingdom) for 2 h at 60°C. After this process, pellets were left to cool at room temperature, and subsequently the essential amino acids were mixed with fish oil fraction according to each target formulation and added under vacuum coating conditions in a Pegasus vacuum mixer (PG-10VCLAB, DINNISEN, The Netherland).

**Table 1:** Ingredients of the experimental diets.

	**Experimental diets**				
**Ingredients (% feed basis)**	**CTRL**	**ARG1**	**CIT1**	**ARG2**	**CIT2**
Fishmeal Super Prime (Diamante) ^a^	5.00	5.00	5.00	5.00	5.00
Hemoglobin powder ^b^	2.00	2.00	2.00	2.00	2.00
Poultry meal 65 ^c^	5.00	5.00	5.00	5.00	5.00
Wheat gluten ^d^	17.00	17.00	17.00	17.00	17.00
Corn gluten ^e^	30.00	30.00	30.00	30.00	30.00
Rapeseed meal ^f^	5.00	5.00	5.00	5.00	5.00
Corn meal ^g^	12.95	12.95	12.95	12.95	12.95
Fish oil ^h^	14.50	14.50	14.50	14.50	14.50
Vit & Min Premix PV01 ^i^	1.00	1.00	1.00	1.00	1.00
Soy lecithin ^j^	1.00	1.00	1.00	1.00	1.00
Binder ^k^	1.00	1.00	1.00	1.00	1.00
Antioxidant powder ^l^	0.20	0.20	0.20	0.20	0.20
Sodium propionate ^m^	0.10	0.10	0.10	0.10	0.10
MCP ^n^	3.00	3.00	3.00	3.00	3.00
L-Arginine°		0.50		1.00	
L-Citrulline ^p^			0.50		1.00
L-Histidine ^q^	0.20	0.20	0.20	0.20	0.20
L-Lysine ^r^	1.50	1.50	1.50	1.50	1.50
L-Threonine ^s^	0.30	0.30	0.30	0.30	0.30
L-Tryptophan ^t^	0.15	0.15	0.15	0.15	0.15
DL-Methionine ^u^	0.10	0.10	0.10	0.10	0.10
**Proximate analyses**					
Dry matter (% feed)	91.61	91.79	92.54	91.94	93.15
Crude protein (% dry weight)	46.91	47.19	47.61	47.33	48.08
Crude lipid (% dry weight)	16.72	17.01	16.94	17.01	16.96
Ash (% dry weight)	5.81	5.85	5.75	5.77	5.78
Gross Energy (kJ g^−1^ DM)	21.57	21.52	21.79	21.66	21.89

a Fish meal Super Prime: 66.3%CP, 11.5% Pesquera Diamante, Peru

b Porcine hemoglobin powder: 91%CP, 1% CF, SONAC BV, The Netherlands

c *Poultry meal: 65%CP, 14.4% CF, SAVINOR UTS, Portugal*.

d *Wheat gluten: 80.4% CP; 5.6% CF, VITAL Roquette, France*.

e *Corn gluten meal: 61% CP, 6% CF, COPAM, Portugal*.

f *Rapeseed meal: Defatted rapeseed meal: 37.7% CP, 2.3% CF, Premix Lda, Portugal*.

g *Corn meal: 10% CP, 4% CF, Ribeiro e Sousa Lda, Portugal*.

h *Fish oil: SAVINOR UTS, Portugal*.

i *Vitamin and mineral premix: PREMIX Lda, Portugal: Vitamins (IU or mg/kg diet): DL-alpha tocopherol acetate, 100 mg; sodium menadione bisulphate, 25 mg; retinyl acetate, 20000 IU; DL-cholecalciferol, 2000 IU; thiamin, 30 mg; riboflavin, 30 mg; pyridoxine, 20 mg; cyanocobalamin, 0.1mg; nicotinic acid, 200 mg; folic acid, 15 mg; ascorbic acid, 500 mg; inositol, 500 mg; biotin, 3 mg; calcium panthotenate, 100mg; choline chloride, 1,000m g, betaine, 500 mg. Minerals (g or mg/kg diet): copper sulfate, 9 mg; ferric sulfate, 6 mg; potassium iodide, 0.5 mg; manganese oxide, 9.6 mg; sodium selenite, 0.01 mg; zinc sulfate,7.5 mg; sodium chloride, 400 mg; excipient wheat middlings*.

j *Soybean lecithin: P700IPM, Lecico GmbH, Germany*.

k *Binder: Kieselguhr (natural zeolite), LIGRANA GmbH, Germany*.

l *Antioxidant: VERDILOX, Kemin Europe NV, Belgium*.

m *Sodium propionate: Disproquímica, Portugal*.

n *Monocalcium phosphate: ALIPHOS MONOCAL, Belgium*.

° *L-Arginine: L-Arginine 95%, Premix Lda, Portugal*.

p *L-Citrulline: L-Citrulline fermentative, Denk, Germany*.

q *L-Hisdidine: L-Histidine 98%, Ajinomoto Eurolysine SAS, France*.

r *L-Lysine: L-Lysine HCl 99%: Ajinomoto Eurolysine SAS, France*.

s *L-Threonine: ThreAMINO 98.5%, Evonik Nutrition & Care GmbH, Germany*.

t *L-Tryptophan: TrypAMINO 98%, Evonik Nutrition & Care GmbH, Germany*.

u *DL-Methionine: DL-METHIONINE FOR AQUACULTURE 99%, EVONIK Nutrition & Care GmbH, Germany*.

**Table 2:** Amino acid composition (g amino acid 100 g^−1^ diet) of the experimental diets.

	**Experimental diets**				
	**CTRL**	**ARG1**	**ARG2**	**CIT1**	**CIT2**
Arginine	3.52	3.96	5.12	3.42	3.52
Citrulline	0.0174	^*^	^*^	0.39	0.81
Histidine	1.08	1.06	1.13	1.02	1.04
Isoleucine	1.68	1.69	1.77	1.70	1.76
Leucine	4.99	5.21	5.06	5.07	5.17
Lysine	2.32	2.31	2.35	2.16	2.20
Threonine	1.65	1.61	1.65	1.67	1.67
Valine	1.99	2.06	2.08	2.05	2.09
Methionine	1.05	1.05	1.04	1.06	1.09
Cystine	0.34	0.34	0.36	0.31	0.33
Methionine + Cystine	1.39	1.39	1.41	1.37	1.42
Phenylalanine	2.22	2.16	2.35	2.27	2.31
Tyrosine	1.77	1.86	1.97	1.79	1.82
Phenylalanine + Tyrosine	3.99	4.02	4.32	4.05	4.14
Taurine	0.04	0.05	0.04	0.03	0.03
Aspartic acid + Asparagine	2.36	2.37	2.39	2.32	2.42
Glutamic acid + Glutamine	10.15	10.01	10.06	9.95	10.12
Alanine	2.36	2.48	2.54	2.46	2.55
Glycine	1.71	1.67	1.76	1.62	1.66
Proline	3.79	3.78	3.84	3.62	3.70
Serine	2.15	2.10	2.11	2.00	2.04

* *not measured, assumed to be the same as the CTRL*.

### Feeding Trial

Samplings were performed at the end of 2 and 4 weeks of feeding in order to assess the effect of short and mid-term dietary supplementation of these amino acids. Feed intake was recorded daily and body weight was measured before the trial and at each sampling point. Growth was monitored by taking the initial body weight (IBW) and final body weight (FBW) in each sampling point.

At the end of each feeding period, four fish from each dietary replicate (12 fish per dietary group) were sacrificed by anesthetic overdose with clove oil and individually weighed. Blood was collected from the caudal vein using heparinized syringes, centrifuged at 10,000 × *g* for 10 min at 4°C and plasma pools were stored at −80°C. Blood was also used to assess blood smears. Head-kidney were also obtained, snap frozen in liquid nitrogen, and stored at −80°C for gene expression.

### Hematological Procedures

Blood smears were firstly fixed with formol-ethanol (10% of 37% formaldehyde in absolute ethanol) and afterwards stained with Wright's stain (Haemacolor; Merck). Neutrophils were identified according to their peroxidase activity, which was detected using the method described by Afonso et al. (23). The slides were examined under oil immersion (1,000 ×) and at least 200 leucocytes were counted and classified as thrombocytes, lymphocytes, monocytes, and neutrophils (24). Each cell type relative proportion was subsequently calculated.

### Innate Immune Parameters

The anti-protease activity was determined as described by Ellis (25) adapted by Machado et al. (24). Briefly, 10 μl of plasma was incubated with the same volume of trypsin solution (5 mg ml^−1^ in NaHCO_3_, 5 mg ml^−1^, pH 8.3) for 10 min at 22°C in polystyrene microtubes. To the incubation mixture, 100 μl of phosphate buffer (NaH_2_PO_4_, 13.9 mg ml^−1^, pH 7.0) and 125 μl of azocasein (20 mg ml^−1^ in NaHCO_3_, 5 mg ml^−1^, pH 8.3) were added and incubated for 1 h at 22°C. Finally, 250 μl of trichloroacetic acid was added to each microtube and incubated for 30 min at 22°C. The mixture was centrifuged at 10,000 × *g* for 5 min at room temperature. Afterwards, 100 μl of the supernatant was transferred in duplicates to a 96 well-plate that previously contained 100 μl of NaOH (40 mg ml^−1^) per well. The OD was read at 450 nm. Phosphate buffer was added to some wells instead of plasma and trypsin and served as blank, whereas the reference sample was phosphate buffer instead of plasma. The percentage of inhibition of trypsin activity compared to the reference sample was calculated.

Total plasma nitrite and nitrate content was measured using a Nitrate/Nitrite colorimetric kit (Roche Diagnostics GmbH, Mannheim, Germany) by adapting it to a 96-well plate and by following manufacturer's instructions. Since both these compounds are derivatives of endogenously produced NO, they are indicative of NO amount in plasma. Briefly, 10 μl of plasma was diluted in 90 μl of distilled water in duplicate to which was then added 50 μl of reduced nicotinamide adenine dinucleotide phosphate (NADPH) and 4 μl of nitrate reductase. A blank was determined by adding distilled water instead of plasma. Absorbance at 540 nm was read after 30 min incubation at 25°C. Afterwards, 50 μl of sulfanilamide and an equal volume of N-(1-naphthyl)-ethylenediamine dihydrochloride were added to each well. The mixture was allowed to stand at 25°C for 15 min and absorbance was read at 540 nm. Total nitrite levels were calculated from a previously prepared sodium nitrite standard curve.

Plasma bactericidal activity was measured according to Graham et al. (26) adapted by Machado et al. (24), with some modifications. Succinctly, 20 μl of plasma was added to duplicate wells of a U-shaped 96-well plate. Hanks' Balanced Salt solution (HBSS) was added to some wells instead of plasma and served as positive control. To each well, 20 μl of *Vibrio anguillarum* (1 × 10^6^ cfu ml^−1^) was added and the plate was incubated for 3 h at 25°C. To each well, 25 μl of iodonitrotetrazolium chloride, INT (2-(4-iodophenyl)-3-(4-nitrophenyl)-5-phenyl-2H-tetrazolium chloride; 1 mg ml^−1^; Sigma) were added to allow the formation of formazan. Plates were then centrifuged at 2,000 × *g* for 10 min and the precipitate was dissolved in 200 μl of dimethyl sulfoxide (Sigma). The absorbance of the dissolved formazan was measured at 490 nm in a Synergy HT microplate reader (Biotek). Total bactericidal activity is expressed as the percentage of killed bacteria, calculated from the difference between the samples (surviving bacteria) and the positive control (100% living bacteria).

Plasma Immunoglobulin M (IgM) was measured by an ELISA assay. Briefly, 4 μl of plasma was previously diluted (1:100) in 396 μl of Na_2_CO_3_ buffer (50 mM, pH = 9.6) and 100 μl of the diluted plasma was added to the 96 wells plate in duplicates. 100 μl of Na_2_CO_3_ buffer was used as a negative control. Samples (antigen) were allowed to adhere to the plate at 22°C for 1 h, and afterwards the samples were removed by means of an aspirator. 300 μl of blocking buffer [5% low fat milk powder in T-TBS (0.1% Tween 20)] was added to each well and left to stand for 1 h incubation period at 22°C. Blocking buffer was then removed by aspiration and wells were washed thrice with 300 μl of T-TBS (0.1% Tween 20). After properly cleaned and dried, 100 μl of the anti-gilthead seabream primary IgM monoclonal antibody previously diluted in blocking buffer (1:100) was added to each well followed by 1 h incubation at 22°C. After removing the primary antibody by aspiration and having washed the wells thrice, 100 μl of the anti-mouse IgG-HRP secondary antibody diluted in blocking buffer (1:1,000) was added to the wells and plate was incubated for 1 h at 22°C. 100 μl of previously prepared TMB substrate solution for ELISA was added to each well after the plate had been aspirated and washed, and plates were incubated for 5 min. The color change reaction was stopped after 5 min by adding 100 μl of 2 M sulphuric acid and the optical density was read at 450 nm.

### Plasma Proteomics

Protein identification and quantitation was performed by nanoLC-MS/MS. This equipment is composed by an Ultimate 3000 liquid chromatography system coupled to a Q-Exactive Hybrid Quadrupole-Orbitrap mass spectrometer (Thermo Scientific, Bremen, Germany). Samples were loaded onto a trapping cartridge (Acclaim PepMap C18 100Å, 5 mm x 300 μm i.d., 160454, Thermo Scientific) in a mobile phase of 2% acetonitrile (ACN), 0.1% formic acid (FA) at 10 μl min^−1^. After 3 min loading, the trap column was switched in-line to a 50 cm by 75 μm inner diameter EASY-Spray column (ES803, PepMap RSLC, C18, 2 μm, Thermo Scientific, Bremen, Germany) at 300 nl min^−1^. Separation was generated by mixing A: 0.1% FA, and B: 80% ACN, with the following gradient: 5 min (2.5% B to 10% B), 120 min (10% B to 30% B), 20 min (30% B to 50% B), 5 min (50% B to 99% B), and 10 min (hold 99% B). Subsequently, the column was equilibrated with 2.5% B for 17 min. Data acquisition was controlled by Xcalibur 4.0 and Tune 2.9 software (Thermo Scientific, Bremen, Germany).

The mass spectrometer was operated in data-dependent (dd) positive acquisition mode alternating between a full scan (m/z 380-1580) and subsequent HCD MS/MS of the 10 most intense peaks from full scan (normalized collision energy of 27%). ESI spray voltage was 1.9 kV. Global settings: use lock masses best (m/z 445.12003), lock mass injection Full MS, chrom. peak width (FWHM) 15 s. Full scan settings: 70 k resolution (m/z 200), AGC target 3e6, maximum injection time 120 ms. dd settings: minimum AGC target 8e3, intensity threshold 7.3e4, charge exclusion: unassigned, 1, 8, >8, peptide match preferred, exclude isotopes on, dynamic exclusion 45s. MS2 settings: microscans 1, resolution 35k (m/z 200), AGC target 2e5, maximum injection time 110 ms, isolation window 2.0 m/z, isolation offset 0.0 m/z, spectrum data type profile.

The raw data was processed using Proteome Discoverer software (Thermo Scientific) and searched against a database for *Sparus aurata* provided by Pauletto et al. (27), deposited in (http://biocluster.her.hcmr.gr/myGenomeBrowser?portalname = Saurata_v1). The Sequest HT search engine was used to identify tryptic peptides. The ion mass tolerance was 10 ppm for precursor ions and 0.02 Da for fragmentations. Maximum allowed missing cleavage sites was 2. Cysteine carbamidomethylation was defined as constant modification. Methionine oxidation and protein N-terminus acetylation were defined as variable modifications. Peptide confidence was set to high. The processing node Percolator was enabled with the following settings: maximum delta Cn 0.05; decoy database search target FDR 1%, validation based on q-value. Protein label free quantitation was performed with the Minora feature detector node at the processing step. Precursor ions quantification was performing at the processing step with the following parameters: Peptides to use unique plus razor, precursor abundance was based on intensity, normalization mode was based on total peptide amount, pairwise protein ratio calculation, hypothesis test was based on *t*-test (background based).

The software PANNZER2 (Protein ANNotation with Z-scorRE) was used to fully automatically annotate the unknown protein functions (28).

### Gene Expression

Total RNA isolation of head-kidney was conducted with NZY Total RNA Isolation kit (NZYTech, Lisbon, Portugal) following manufacturer's specifications. First-strand cDNA was synthesized from a total RNA per sample (280 ng), which was performed using the NZY First-Strand cDNA Synthesis Kit (NZYTech, Lisbon, Portugal). Quantitative PCR assays were performed with an Eppendorf Mastercycle ep realplex, using 1 μl of diluted cDNA (1:5 dilution) mixed with 10 μl of NZYSpeedy qPCR Master Mix and 0.4 μl (10 μM) of each specific primer in a final volume of 20 μl. cDNA amplification was carried out with specific primers for genes that have been selected for their involvement in immune responses and arginine metabolism. Primers were designed with NCBI Primer Blast Tool according to known qPCR restrictions (amplicon size, Tm difference between primers, GC content and self-dimer or cross-dimer formation). Efficiency of primer pairs was analyzed in serial, 5-fold dilutions of cDNA by calculating the slope of the regression line of the cycle thresholds (Ct) vs. the relative concentration of cDNA. Accession number, efficiency values, annealing temperature, product length, and primers sequences are presented in Table 3. Melting curve analysis was also performed to verify that no primer dimers were amplified. The standard cycling conditions were 94°C initial denaturation for 2 min, followed by 40 cycles of 94°C denaturation for 30 s, primer annealing temperature for 30 s and 72°C extension for 30 s. All reactions were carried out as technical duplicates. The expression of the target genes was normalized using the expression of gilthead seabream elongation factor 1 α (*ef1*α).

**Table 3:** Immune-related genes analyzed by real-time PCR.

**Acronym**	**Gene Bank ID**	**Eff^a^**	**AT^b^**	**Product lenght^c^**	**Forward primer sequence**
*il-10*	EF625901	134.62	57	65	**F:** AACATCCTGGGCTTCTATCTG
					**R:** GTGTCCTCCGTCTCATCTG
*il-34*	JX976629.1	96.70	60	214	**F:** CATCAGGGTTCATCACAACG
					**R:** GACTCCCTCTGCATCCTTGA
*il4-13*	MG816480.1	91.77	60	68	**F:** GCTGAGAAGTCCCTGGAAGCACACAATA
					**R:** TACCGACACAAGTGACGAGTAAGGTTTGAT
*il-1β*	AJ277166.2	81.17	60	245	**F:** TCTTCAAATTCCTGCCACCA
					**R:** CAATGCCACCTTGTGGTGAT
*cd8α*	AJ878605	115.44	60	287	**F:** CTCGACTGGTCGGAGTTAA
					**R:** TCCATCAGCGGCTGCTCGT
*cd4*	AM489485.1	80.56	60	60	**F:** TCCTCCTCCTCGTCCTCGTT
					**R:** GGTGTCTCATCTTCCGCTGTCT
*tnf-α*	AJ413189.2	104.87	60	245	**F:** TGAACAGAGGCGACAAACTG
					**R:** GCCACAAGCGTTATCTCCAT
*IgM*	AM493677	115.44	59	136	**F:** CAGCCTCGAGAAGTGGAAAC
					**R:** GAGGTTGACCAGGTTGGTGT
*tcrβ*	AM261210	93.07	59	131	**F:** AAGTGCATTGCCAGCTTCTT
					**R:** TTGGCGGTCTGACTTCTCTT
*csfr*	AM050293	127.58	60	129	**F:** ACGTCTGGTCCTATGGCATC
					**R:** AGTCTGGTTGGGACATCTGG
*tgfβ2*	AM749962	92.54	60	127	**F:** GAGCAGGGCTTTGAGACAGT
					**R:** CTGTCAGGAAGTGGAGCACA
*arg-II*	XM_030443793.1	93.39	60	82	**F:** TGGAACGCCAGTCAACGGA
					**R:** CGACAGCAGACCTGTGTTATGGA
ef1α^*^	AF184170	105.35	58	87	**F:** CTGTCAAGGAAATCCGTCGT
					**R:** TGACCTGAGCGTTGAAGTTG

a *Efficiency of PCR reactions were calculated from serial dilutions of tissue RT reactions in the validation procedure*.

b *Annealing temperature (°C)*.

c *Amplicon (nt)*.

### Plasma Proteomics

**Figure 1:**
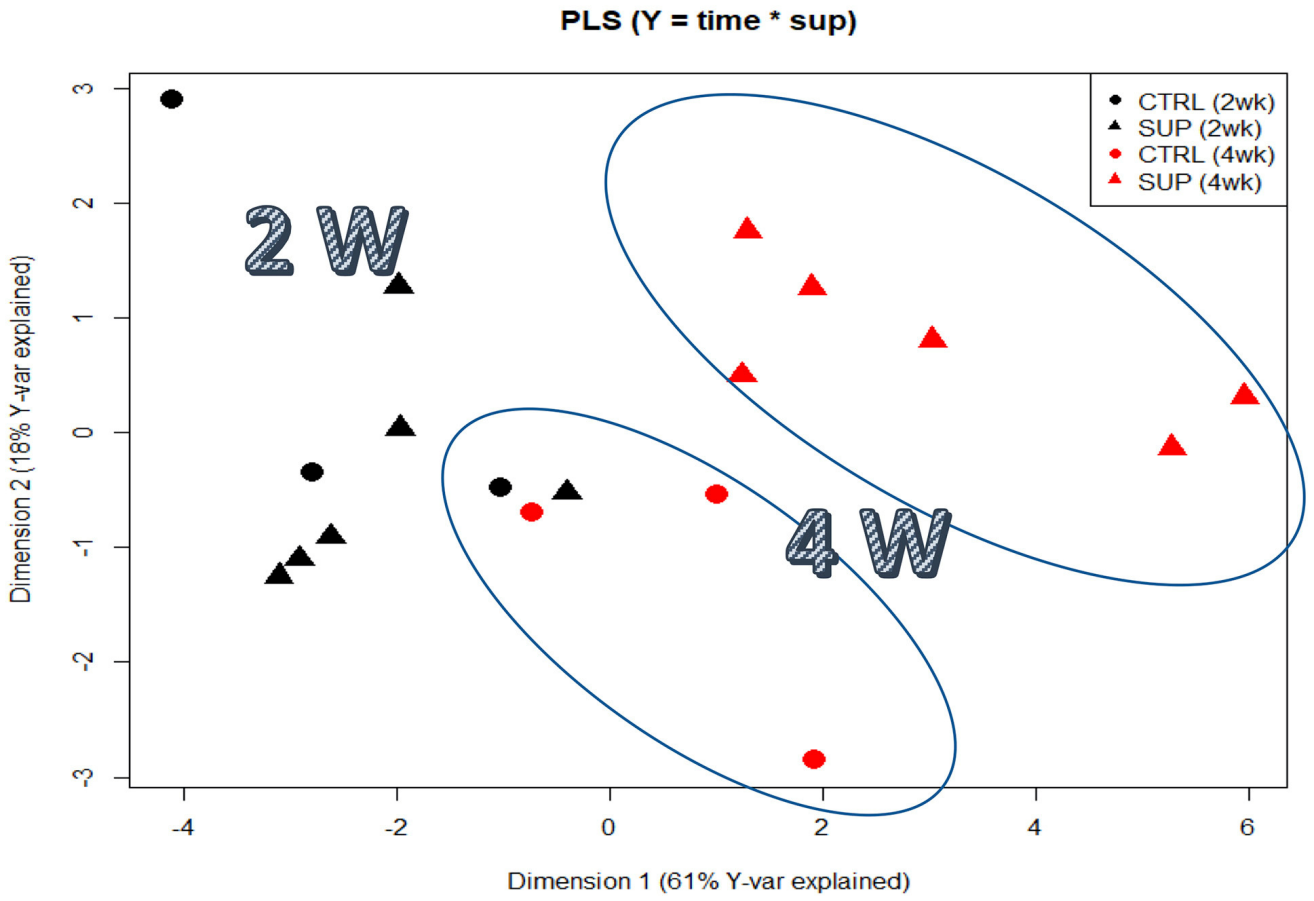
Partial least squares regression analysis (PLSR) of plasma proteomic signatures of fishes fed the experimental diets. PLS score plots of all proteins analyzed along the two main components, considering all supplemented diets as a pool designated as SUP. The Y-matrix contained factors “time,” “supplemented” and the interaction between these two factors.

A total of 92 proteins were identified in the plasma of all treatments and a detailed list is provided in Table S1. Among them, components of the complement system, apolipoproteins, as well as some glycoproteins were found to be highly abundant (Figure S1). The PLSR analysis of the expressed proteins showed differences between the two sampling points independently of dietary treatment. In this regard, component 1 (61%) explained the effect of sampling time, whereas the interpretation of component 2 (18%) is not as clear. Nonetheless, it can be observed that the plasma proteome profile of fish fed the supplemental diets is seemingly affected after 4 weeks of feeding, compared to fish fed CTRL (Figure 1). Overall, only 19 out of 92 proteins were significantly modulated in fish fed diets with the highest inclusion of arginine and citrulline after 4 weeks, compared to fish fed other treatments for 2 weeks (**Table 7** & Figure S2). Proteins of the complement system (highlighted in green) were highly modulated in fish fed the supplemented diets after 4 weeks compared to fish fed other dietary treatments over a shorter period of 2 weeks, which is in accordance with previous immune-related data analyzed in this trial (**Tables 5**, **6**).

### Gene Expression

**Figure 2:**
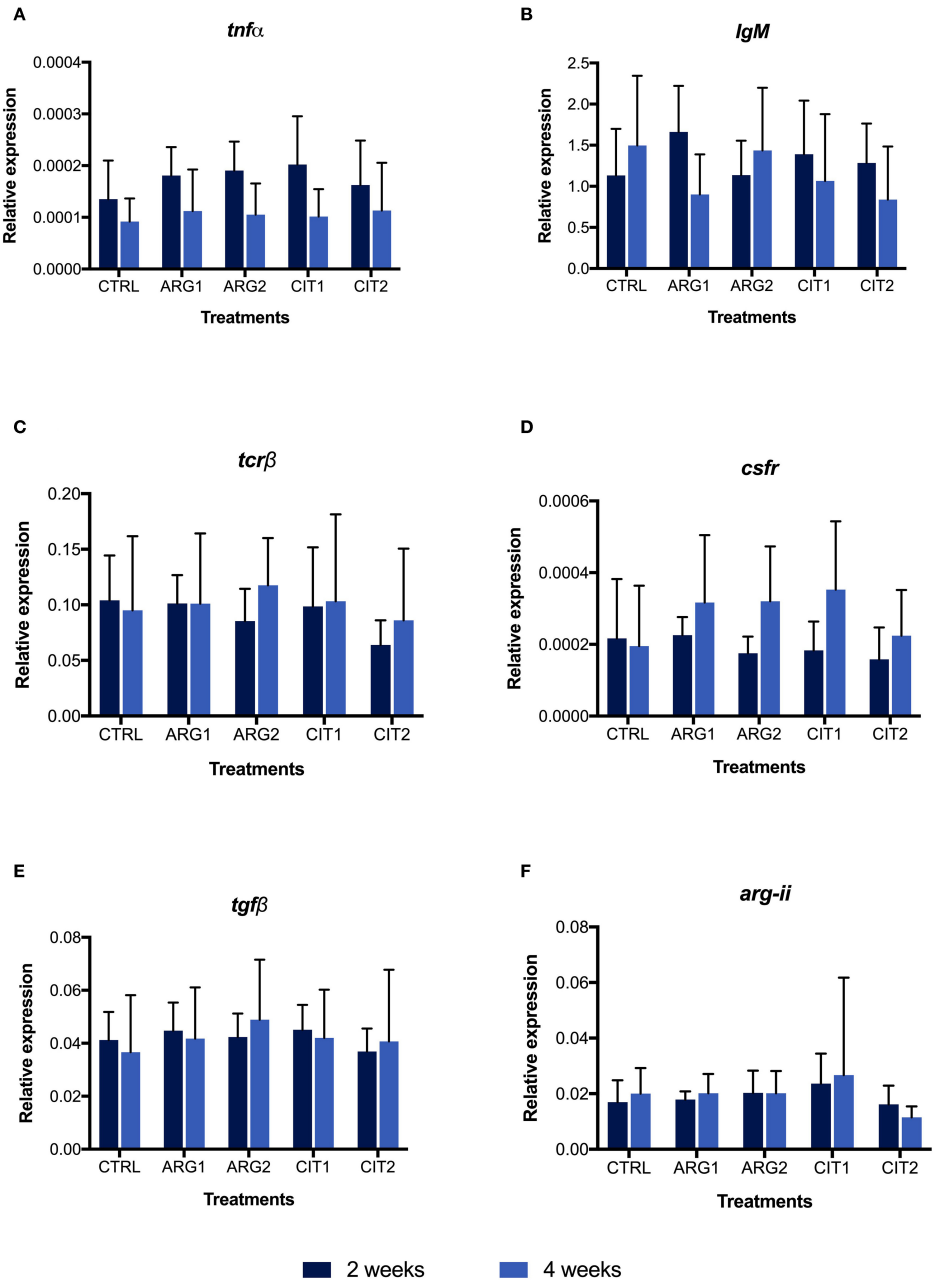
Quantitative expression of tumor necrosis factor α (*tnf*α, **A**), immunoglobulin M (heavy chain) (*IgM*, **B**), T cell receptor β (*tcr*β, **C**), colony stimulating factor-1 receptor (*csfr*, **D**), transforming growth factor beta (*tgf*β, **E**), arginase type II precursor (*arg-ii*, **F**) genes in the head-kidney of gilthead seabream fed dietary treatments during 2 (■) and 4 weeks (■). Values are presented as means ± SD (*n* = 9). *P*-values from Two-way ANOVA (*p* ≤ 0.05). Tukey *post-hoc* test was used to identify differences in the experimental treatments. Different lowercase letters stand for significant differences among dietary treatments for the same time. Different capital letters indicate differences among diets regardless time. Different symbols indicate difference among time for the same dietary treatment.

**Figure 3:**
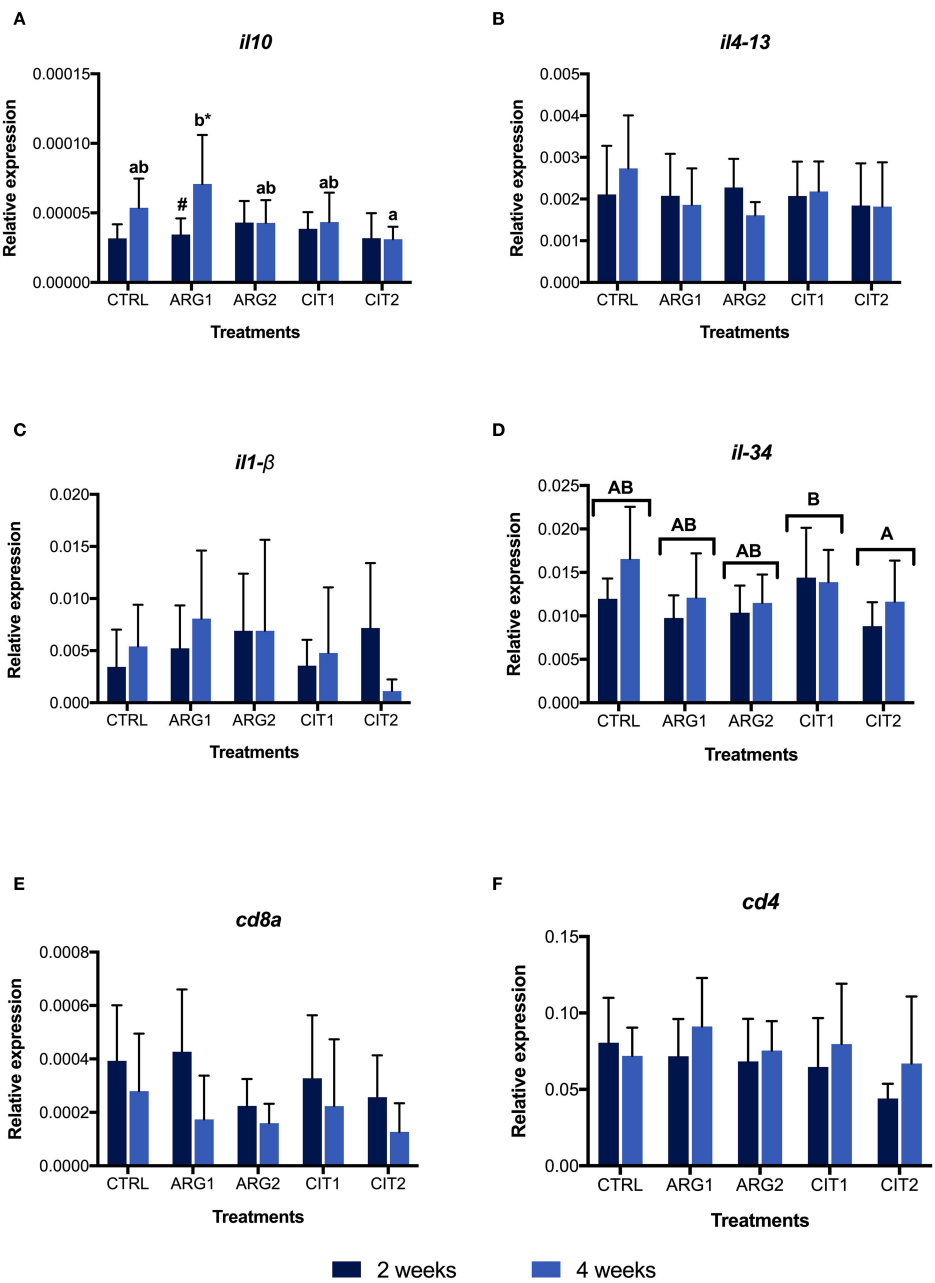
Relative expression of interleukin 1 β (*il1-*β, **A**), interleukin 4-13b (*il4-13* **B**), interleukin 10 (*il-10*, **C**), interleukin 34 **(***il-34*, **D**), cluster of differentiation 8α (*cd8*α, **E**) cluster of differentiation 4 (*cd4*, **F**) genes in the head-kidney of gilthead seabream fed dietary treatments during 2 (■) and 4 weeks (■). Values are presented as means ± SD (*n* = 9). *P*-values from two-way ANOVA (*p* ≤ 0.05). Tukey *post-hoc* test was used to identify differences in the experimental treatments. Different lowercase letters stand for significant differences among dietary treatments ≤ for the same time. Different capital letters indicate differences among diets regardless time. Different symbols indicate difference among time for the same dietary treatment.

**Figure 4:**
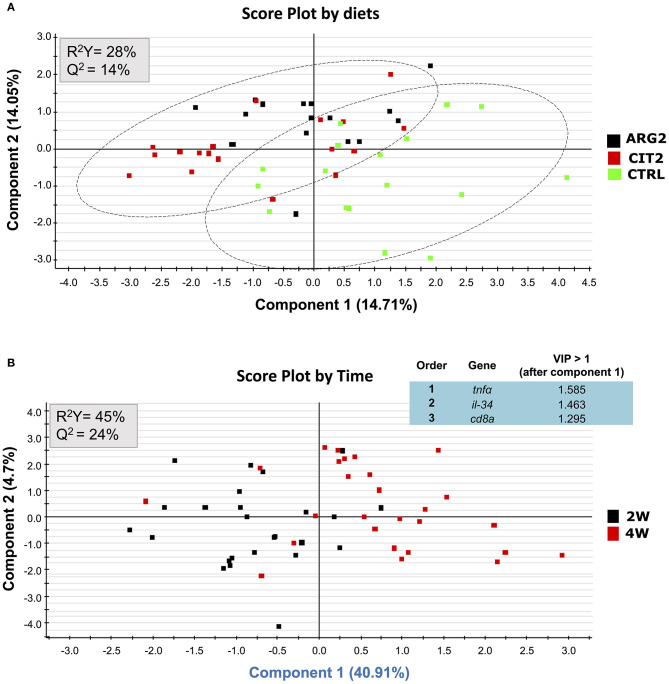
Discriminant analyses (PLS-DA) of head-kidney molecular signatures of gilthead seabream fed the experimental diets. Relative expression data of the 12 biomarkers can be found on Table S2. **(A)** PLS-DA score plots of all biomarkers analyzed in groups fed the highest supplementation level and the control, along the two main components, where the analysis was focused on the “diet” factor. **(B)** PLS-DA score plots of all biomarkers analyzed along the two main components, where the analysis was focused on the “time” factor and ordered list of markers by variable importance (VIP) in projection of PLS-DA model for time differentiation. Markers with VIP values > 1 after the first component is highlighted in blue.

No differences were observed in gene expression between supplemented diets and the control group, but differences were observed between sampling points (Table S2; Figure 2 and Figures 3A,B,E,F). Nonetheless, the *il-10* transcript was up-regulated in fish fed ARG1 for 4 weeks compared to those fed CIT2 (Figure 3C). Moreover, *il-*34 was up-regulated in fish fed CIT1 when compared to CIT2 regardless of sampling time (Figure 3D). Besides the classical ANOVA analysis approach, an overall multivariate analysis (using PLS-DA) was performed (Figure 4). PLS-DA analysis focused on the “diet” factor suggests that the expression data could not extensively explain all differences between diets (R^2^Y of 28%, Figure 4A), with the obtained prediction capacity being also low (Q^2^ of 14%). Despite this fact, it was observed that, when samples are plotted along the first two components (Figure 4A), supplemented samples tend to lie closer to the upper-left quadrant, while non-supplemented samples lie closer to the bottom-right quadrant. When performing the PLS-DA analysis focusing on the “time” factor (Figure 4B), it was able to explain 45% of Y-variance R^2^Y and to predict more than 24% of the total variance (Q^2^). In this particular analysis, component 1 represented sampling time effects (C1, 40.9%), whereas the interpretation of component 2 (4.6%) was not clear (Figure 4B), thus confirming a clear time effect on the biomarkers panel analyzed. In order to understand and interpret the contribution of the different genes to these components, a table of the variable importance projection (VIP) score of the genes ordered by its importance is presented in Figure 4B. It is clear that all biomarkers were highly affected by sampling time (Component 1, highlighted in blue).

## Results

### Growth Performance

No differences were observed in final body weight (FBW), daily growth index (DGI), relative growth rate (RGR), voluntary feed intake (VF), feed efficiency (FE), or feed conversion ratio (FCR) among the dietary treatments (Table 4).

**Table 4:** Body weight (BW, g fish^−1^), Relative growth rate (RGR), daily growth index (DGI), feed conversion ratio (FCR), feed efficiency (FE), and voluntary feed intake (VFI) of gilthead seabream fed the experimental diets for 2 and 4 weeks.

		**CTRL**		**ARG1**		**ARG2**		**CIT1**		**CIT2**	
		**2 weeks**	**4 weeks**	**2 weeks**	**4 weeks**	**2 weeks**	**4 weeks**	**2 weeks**	**4 weeks**	**2 weeks**	**4 weeks**
BW	(g)	26.58 ± 1.65	35.34 ± 1.41	26.92 ± 0.29	36.32 ± 0.48	27.70 ± 0.69	36.31 ± 1.93	26.55 ± 0.73	36.37 ± 2.75	28.23 ± 1.01	34.46 ± 2.15
RGR	(% day^−1^)	0.97 ± 0.33	1.46 ± 0.17	1.16 ± 0.06	1.60 ± 0.04	1.32 ± 0.28	1.58 ± 0.17	0.95 ± 0.22	1.55 ± 0.29	1.49 ± 0.24	1.41 ± 0.16
DGI	(% day^−1^)	0.94 ± 0.34	1.48 ± 0.16	1.11 ± 0.06	1.62 ± 0.04	1.29 ± 0.27	1.60 ± 0.19	0.92 ± 0.21	1.58 ± 0.31	1.45 ± 0.24	1.42 ± 0.18
FCR		2.63 ± 0.91	1.79 ± 0.20	2.01 ± 0.11	1.73 ± 0.05	1.78 ± 0.37	1.71 ± 0.12	2.56 ± 0.56	1.84 ± 0.25	1.56 ± 0.31	1.85 ± 0.11
FE		0.41 ± 0.15	0.56 ± 0.07	0.50 ± 0.03	0.58 ± 0.02	0.58 ± 0.13	0.58 ± 0.04	0.41 ± 0.10	0.55 ± 0.08	0.66 ± 0.12	0.54 ± 0.03
VFI	(% BW)	2.33 ± 0.06	2.93 ± 0.06	2.30 ± 0.01	2.97 ± 0.02	2.27 ± 0.05	2.96 ± 0.06	2.33 ± 0.04	2.95 ± 0.10	2.24 ± 0.04	2.91 ± 0.06

### Peripheral Blood Leucocytes

**Figure 5:**
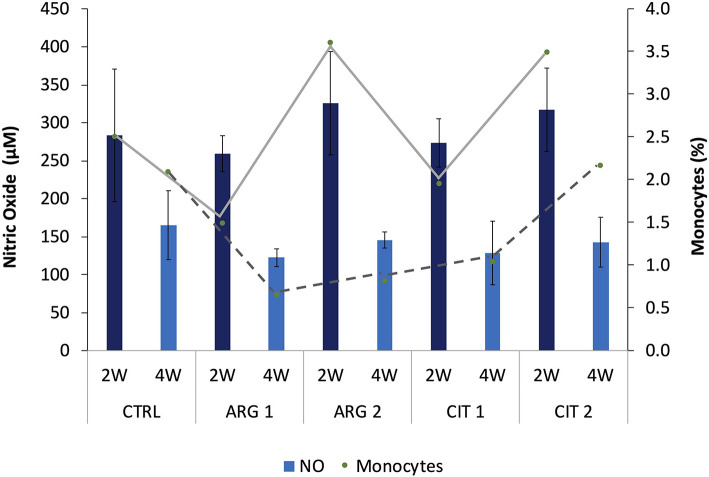
Nitric oxide levels and monocytes relative values (trend lines) of gilthead seabream fed dietary treatments during the feeding trial are positively correlated (*R*^2^ = 0.339, *p* < 0.001). Values are presented as means ± SD (*n* = 9).

With the exception of monocytes, the relative proportion of peripheral blood leucocytes (thrombocytes, lymphocytes and neutrophils) of gilthead seabream fed dietary treatments during 2 and 4 weeks were similar among dietary treatments and sampling times (Table 5). The proportion of monocytes was observed to be higher in fish fed diets with the highest supplementation level (i.e., ARG2 and CIT2) compared to their respective counterparts fed the lower supplementation level (Figure 5). Overall, fish fed CIT2 had the highest relative proportion of circulating monocytes regardless of sampling point.

**Table 5:** Relative proportion of peripheral blood leucocytes (thrombocytes, lymphocytes, monocytes and neutrophils) of gilthead seabream fed dietary treatments during 2 and 4 weeks.

**Parameters**		**CTRL**		**ARG1**		**ARG2**		**CIT1**		**CIT2**	
		**2 weeks**	**4 weeks**	**2 weeks**	**4 weeks**	**2 weeks**	**4 weeks**	**2 weeks**	**4 weeks**	**2 weeks**	**4 weeks**
Thrombocytes	(%)	57.94 ± 6.97	64.73 ± 9.09	57.83 ± 7.87	61.63 ± 12.95	61.44 ± 8.73	69.63 ± 10.71	60.89 ± 8.80	66.24 ± 10.31	55.77 ± 5.67	62.33 ± 13.63
Lymphocytes	(%)	30.51 ± 6.96	26.36 ± 7.60	32.89 ± 9.24	31.48 ± 10.42	29.65 ± 6.47	24.38 ± 9.24	29.80 ± 8.71	26.05 ± 7.92	31.38 ± 8.33	25.47 ± 13.49
Monocytes	(%)	2.50 ± 2.37	2.09 ± 1.69	1.49 ± 0.78	0.65 ± 0.67	3.61 ± 2.50	0.81 ± 1.53	1.95 ± 1.48	1.04 ± 0.96	3.50 ± 1.69	2.17 ± 2.28
Neutrophils	(%)	9.04 ± 5.07	6.81 ± 3.95	7.78 ± 5.62	6.25 ± 4.11	5.31 ± 3.10	5.19 ± 4.96	7.36 ± 3.07	6.67 ± 5.75	9.36 ± 4.76	10.03 ± 7.14
**Two-way ANOVA**											
**Parameters**					**Time**		**Diet**				
		**Time**	**Diet**	**Time x diet**	**2 Weeks**	**4 Weeks**	**CTRL**	**ARG1**	**ARG2**	**CIT1**	**CIT2**
Thrombocytes	(%)	0.002	0.188	0.963	A	B	-	-	-	-	-
Lymphocytes	(%)	0.021	0.414	0.943	B	A	-	-	-	-	-
Monocytes	(%)	<0.001	0.014	0.236	B	A	AB	A	AB	AB	B
Neutrophils	(%)	0.411	0.072	0.883	-	-	-	-	-	-	-

*Values are presented as means ± SD (n = 9). P-values from Two-way ANOVA (p ≤ 0.05). Tukey post-hoc test was used to identify differences in the experimental treatments. Different capital letters indicate differences between diets regardless of time or difference between times regardless of diets*.

### Immune Parameters in Plasma

Bactericidal activity increased in fish fed ARG2 compared to fish fed the CTRL diet after 4 weeks of feeding (Table 6). Plasma NO levels were enhanced in fish fed the highest supplementation levels regardless of sampling time (Table 6). A decrease in antiprotease activity was observed in dish fed ARG2 from the first to final sampling point. No differences were observed in IgM levels (Table 6). Moreover, monocytes from fish fed ARG2 and CIT2 were also found to be positively correlated with NO levels measured in plasma and showed and increasing trend in a dose-dependent manner (R^2^ = 0.339, *p* < 0.001) (Figure 1).

**Table 6:** Plasma humoral parameters of gilthead seabream fed dietary treatments during 2 and 4 weeks.

**Parameters**		**CTRL**		**ARG1**		**ARG2**		**CIT1**		**CIT2**	
	**2 weeks**	**4 weeks**	**2 weeks**	**4 weeks**	**2 weeks**	**4 weeks**	**2 weeks**	**4 weeks**	**2 weeks**	**4 weeks**	
Antiprotease activity (%)	93.41 ± 1.51	93.65 ± 1.46	94.45 ± 1.04	93.58 ± 2.33	95.22 ± 0.68*	92.16 ± 2.02^#^	95.00 ± 1.36	93.58 ± 1.59	94.80 ± 0.94	92.18 ± 2.81	
Bactericidal activity (%)	62.41 ± 8.03	63.31 ± 6.57^**ab**^	59.18 ± 9.22	61.58 ± 6.19^**a**^	53.70 ± 9.17	71.29 ± 4.84^**c**^	59.46 ± 7.63	69.52 ± 6.40^**bc**^	56.64 ± 11.51^#^	69.92 ± 4.31^bc*^	
IgM (abs)	0.80 ± 0.27	0.91 ± 0.41	0.74 ± 0.30	0.76 ± 0.38	0.69 ± 0.29	0.61 ± 0.24	0.57 ± 0.26	0.73 ± 0.21	0.68 ± 0.25	0.78 ± 0.30	
Nitric oxide (μM)	283.40 ± 87.24	165.17 ± 45.40	259.19 ± 23.73	122.26 ± 11.72	325.70 ± 68.01	145.64 ± 10.59	273.44 ± 31.76	128.59 ± 41.82	317.08 ± 54.86	142.76 ± 32.78	
**Two-way ANOVA**											
**Parameters**					**Time**		**Diet**				
		**Time**	**Diet**	**Time x diet**	**2 Weeks**	**4 Weeks**	**CTRL**	**ARG1**	**ARG2**	**CIT1**	**CIT2**
Antiprotease activity	(%)	<0.001	0.486	0.016	B	A	-	-	-	-	-
Bactericidal activity	(%)	<0.001	0.473	0.002	A	B	-	-	-	-	-
IgM	(abs)	0.261	0.113	0.635	-	-	-	-	-	-	-
Nitric Oxide	(μM)	<0.001	0.016	0.257	B	A	AB	A	B	AB	B

*Values are presented as means ± SD (n = 9). P-values from two-way ANOVA (p ≤ 0.05). Tukey post-hoc test was used to identify differences in the experimental treatments. Different lowercase letters stand for significant differences between dietary treatments for the same time. Different capital letters indicate differences between diets regardless of time or difference between times regardless of diets. Different symbols indicate difference between times for the same dietary treatment*.

### Plasma Proteomics

A total of 92 proteins were identified in the plasma of all treatments and a detailed list is provided in Table S1. Among them, components of the complement system, apolipoproteins, as well as some glycoproteins were found to be highly abundant (Figure S1). The PLSR analysis of the expressed proteins showed differences between the two sampling points independently of dietary treatment. In this regard, component 1 (61%) explained the effect of sampling time, whereas the interpretation of component 2 (18%) is not as clear. Nonetheless, it can be observed that the plasma proteome profile of fish fed the supplemental diets is seemingly affected after 4 weeks of feeding, compared to fish fed CTRL (Figure 2). Overall, only 19 out of 92 proteins were significantly modulated in fish fed diets with the highest inclusion of arginine and citrulline after 4 weeks, compared to fish fed other treatments for 2 weeks (Table 7; Figure S2). Proteins of the complement system (highlighted in green) were highly modulated in fish fed the supplemented diets after 4 weeks compared to fish fed other dietary treatments over a shorter period of 2 weeks, which is in accordance with previous immune-related data analyzed in this trial (Tables 5, 6).

**Table 7:** Differently expressed proteins in fish fed the highest supplementation levels of both ARG and CIT in study after 4 weeks of feeding.

**Accession**	**Description**	**Biological process**	**Molecular function**	***p*-values**
Sa_23268.2.1	Complement component 7a	Complement activation	Chitin binding	0.0037
Sa_4795.1.1	Phosphoglucomutase 1	Carbohydrate metabolic process	Intramolecular transferase activity, phosphotransferases	0.0045
Sa_46414.1.1	Complement component 7b	Complement activation	Chitin binding	0.0078
Sa_9379.3.3	Alpha-1,4 glucan phosphorylase	Carbohydrate metabolic process	Glycogen phosphorylase activity	0.0094
Sa_48024.9.1	Tributyltin binding protein type			0.0157
Sa_18106.7.1	Leucine zipper-EF-hand containing transmembrane protein 1	Ca export from the mitochondrion ribosome binding mitochondrion		0.0171
Sa_24913.2.4	Coagulation factor IXa	Blood coagulation	Calcium ion binding	0.0205
Sa_27134.1.1	Uncharacterized protein	Semaphorin-plexin signaling pathway	Semaphorin receptor activity	0.0226
Sa_13814.1.1	intelectin	Induction of bacterial agglutination	Oligosaccharide binding	0.0246
Sa_33122.3.1	apolipoprotein C-II	Lipid transport	Enzyme activator activity	0.0274
Sa_25136.3.1	Phosphoglycerate mutase	ATP generation from ADP/pyruvate metabolic process	Bisphosphoglycerate mutase activity	0.0282
Sa_26882.2.1	Uncharacterized protein	Complement activation	Endopeptidase inhibitor activity	0.0317
Sa_19875.1.1	Complement factor D	Complement activation, alternative pathway	Serine-type endopeptidase activity	0.0319
Sa_12582.3.1	Sex hormone-binding globulin type-II	Response to estradiol	Androgen binding	0.0410
Sa_32547.2.1	Uncharacterized protein			0.0416
Sa_1942.4.1	Fucose mutarotase	Monosaccharide metabolic process	Monosaccharide binding	0.0417
Sa_44333.2.1	Kininogen	Negative regulation of endopeptidase activity	Cysteine-type endopeptidase inhibitor activity	0.0420
Sa_12688.1.1	Thyroid hormone receptor interactor 10	Vesicle-mediated transport	Lipid binding	0.0423
Sa_41650.2.1	Plasminogen activator inhibitor 2	Negative regulation of fibrinolysis	Serine-type endopeptidase inhibitor activity	0.0441

*The colors stand for the graded level of p-values, greener is lower that light green of yellow*.

## Discussion

Arginine surplus has proven to be a good strategy to modulate both the innate and adaptive immune fish response, where arginine may act through polyamines, directedly by modulating gene expression, by regulating nutrient availability for immune cells through the endocrine control or through NO to fight pathogens (29). The present study represents the first attempt to explore the effects of dietary supplementation of arginine and citrulline on the gilthead seabream immune system. Results showed a light modulation of the immune status after 2 weeks of feeding, mainly in the relative proportion of monocytes, which decreased in time regardless of dietary treatments. Chen et al. (30) observed that in fish fed a diet rich in arginine (up to 21.9 g/kg diet) upon a challenge with *Aeromonas hydrophila* some health-related biomarkers like TNF-α and TGF-β where up-regulated in head-kidney, and decreased in higher inclusion levels, reiterating a dose-dependent effect. This may also point, that arginine supplementation efficacy on immune system, is dependent of an immune stimuli.

Noteworthy, fish fed the highest citrulline level had the highest relative proportion of monocytes regardless of sampling point. Monocytes from fish fed ARG2 and CIT2 were also found to be positively correlated with NO levels measured in plasma and showed an increasing trend in a dose-dependent manner. It is well-known that NO is formed through the oxidation of _L_-arginine in a reaction catalyzed by the inducible NO synthases (iNOS), an enzyme mainly expressed in activated monocytes, macrophages and neutrophils, highlighting the importance of arginine availability upon immune stimulation (31, 32). Moreover, NO plays an important dual role in host defense against pathogens and cytotoxic actions in some pathological processes, acting as both pro-inflammatory or it can promote immunosuppression (33). Interestingly, Rapovy et al. (34) found that arginase-expressing macrophages preferred L-citrulline over L-arginine for the promotion of antimycobacterial activity. Briefly, NO production was not compromised in mice co-stimulated macrophages with *Mycobacterium bovis* and interferon γ, expressing the arginase gene and cultured in citrulline-supplemented media, in contrast to those maintained in arginine-supplemented (34).

Arginine being a major substrate for polyamine biosynthesis, essential for cell proliferation, when in surplus may increase total leukocytes, particularly macrophages-secreted cytokines (35). Hence, these results suggest that the inclusion of either amino acid at the highest level could boost to some extent the gilthead seabream immune response after a short feeding period of 2 weeks. Moreover, seabream fed ARG2 increased plasma bactericidal activity following 4 weeks of feeding. Bactericidal activity is a multifactorial indicator since it evaluates a wide range of innate immune mechanisms and molecular defenses against bacterial invasion, such as proteins of the complement system, acute phase proteins and cytokines (26, 36). Therefore, an increase of this parameter seems to indicate that fish fed ARG2 for 4 weeks have an enhanced immune status and might thereby develop a more efficient immune response.

Li et al. (37) showed that in an *in vitro* study with isolated carp erythrocytes, that the combination of several AA, among them citrulline (Gln, Ala, Cit, and Pro) was able to confer protection to the cultured cells against oxidative damage (e.g., generation of ROS) induced by hydroxyl radical (•OH), leading probably to its protection from apoptosis. This action might be useful during an overproduction of radicals exceeding the antioxidant capacity of a cell.

In general, arginine and citrulline supplementation seemed to induce higher transcriptional changes, compared to the effect of the “time” factor. Taking into account the discriminant analysis (PLS-DA) that integrated the expression data from head-kidney, it can be seen that only data from fish fed the highest AA supplementation revealed a higher capacity to predict “time” instead of predict “diet.”

On the other hand, we consider that the low predicted variance (Q^2^) values observed for the “diet” factor can, at least in part, be explained by the strong similarity between the ARG2 and CIT2 diets, in terms of their effects on the head kidney transcriptome.

High-throughput proteomics are gaining rapid traction in teleost physiology (38), and it is well-known that there should not be an expectation of a direct relationship between head-kidney transcriptomic response and plasma protein abundance. Hence, in order to better understand the modulation capacity of Arginine and Citrulline supplementation, plasma samples were evaluated in terms of their protein content. This approach was very useful to quantify a total of 92 plasma proteins, and particularly those that were modulated by the experimental diets. Among others, many proteins of the complement system, apolipoproteins, as well as some glycoproteins were identified. A PLSR analysis of the expressed proteins showed that, despite “time” having a strong effect on plasma protein expression, a clear separation between fish fed the highest supplementation levels (ARG2 and CIT2) and CTRL was found at 4 weeks, while after a shorter feeding period of 2 weeks no differences were observed between experimental dietary treatments. A notable finding was the modulation of proteins of the complement system by the supplemented amino acids. These proteins are activated through the classical, alternative and lectin pathways, leading to bactericidal actions through pathogen opsonization, phagocytic activity, and subsequent lysis (39, 40). Data from the present study highlighted two complement proteins subunits, C7a and C7b, proteins member of the lytic pathway. Along with C5b to C9, C7 is part of the formation of a porous transmembrane structure that is inserted into the lipid membrane of the microbial agent and causes cytolysis. C7 also possesses short-consensus repeats, tandem structural units that are found in plasma and membrane complement-regulatory proteins (41). Nonetheless, recent evidences suggest that complement proteins functions go beyond the immune role, such as metabolic functions, particularly insulin-like roles and triglyceride metabolism facilitators (39).

Arginine and its metabolites have been described to strongly affect tissue repair and cell replication—processes involved in animal growth and survival (42–44). In the current study, no differences were observed in growth and feed conversion parameters. This is in line with results observed in a previous trial with juvenile red drum, where a diet supplemented with arginine did not alter growth performance (45). However, in that study the same basal diet supplemented with a combination of arginine and glutamine (at the same inclusion level) improved significantly feed efficiency, compared to those fed the basal diet, during a 7-weeks feeding trial (45). This suggests that arginine plays a co-operative role and is able to increment glutamine effect on feed efficiency, rather than a direct effect on fish growth performance. However, the mechanism for the beneficial effect of this combination is still unclear.

Moreover, Liang et al. (46) showed that juvenile blunt snout bream (*Megalobrama amblycephala*) fed an enriched arginine diet for 8 weeks had a better growth performance (optimal FCR, at 1.62% of dry diet), but in a dose-dependent manner, since either a higher or lower inclusion levels had negative impact on fish growth performance. Arginine (at 2.54% of diet) was also reported to improve feed utilization (some improvements on FCR) in a 9-weeks feeding trial with juvenile red seabream (*Pagrus major*) (47). Taking into account that these trials had a longer feeding period, characteristic of a growth trial, the fact that no relevant growth performance-effect was observed in the present study could be time-related.

In summary, the present study unveiled a stimulation of the fish immune status for a short-term feeding period, verified mostly by a modulation of the gilthead seabream plasma proteome and health-biomarkers after 4 weeks of feeding diets rich in arginine and citrulline, particularly when supplemented at a 1% inclusion level. The concomitant increase of plasma bactericidal activity in fish fed ARG2 for the same period was also observed, and the peripheral monocyte numbers correlated positively with nitric oxide, which showed an increasing trend in a dose-dependent manner.

## Data Availability Statement

The raw data supporting the conclusions of this article will be made available by the authors, without undue reservation.

## Ethics Statement

The handling of animals at this experiment complied with the guidelines of the European Union Council (86/609/EU) and Spanish Legislation (RD 53/2013) and was approved by the Bio- ethical Committee of the ULPGC (Ref. 007/2012 CEBA ULPGC).

## Author Contributions

LC, DM, TS, and BC conceived the experiment and contributed with both reagents and goods. CS and ST conducted the experimental feeding trial and collected all samples. LR-P conducted the main experimental work. RA assisted with analytical procedures. LR-P directed most laboratory techniques and wrote the manuscript under the supervision of JD, LC, DM, TS, and BC. All authors contributed to and approved the manuscript.

## Conflict of Interest

The authors declare that the research was conducted in the absence of any commercial or financial relationships that could be construed as a potential conflict of interest.
