# Supplementary material for: Short-Term Supplementation of Dietary Arginine and Citrulline Modulates Gilthead Seabream (Sparus aurata) Immune Status
Source: Front Immunol. 2020 Aug 5;11:1544. doi: 10.3389/fimmu.2020.01544 (PMC7419597; doi:10.3389/fimmu.2020.01544)
Supplement: Supplementary file 1 [file Data_Sheet_1.docx]

**Supplementary Files**


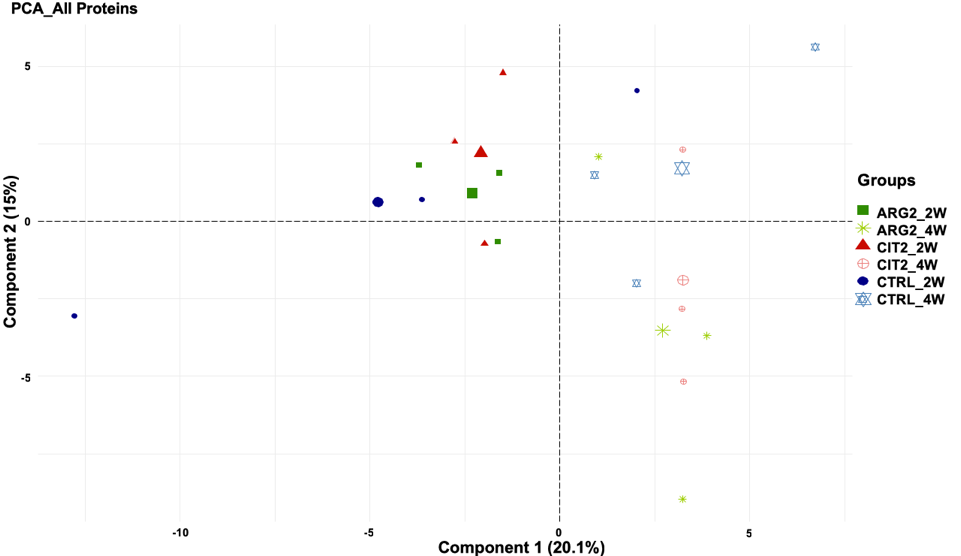


A

B
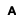


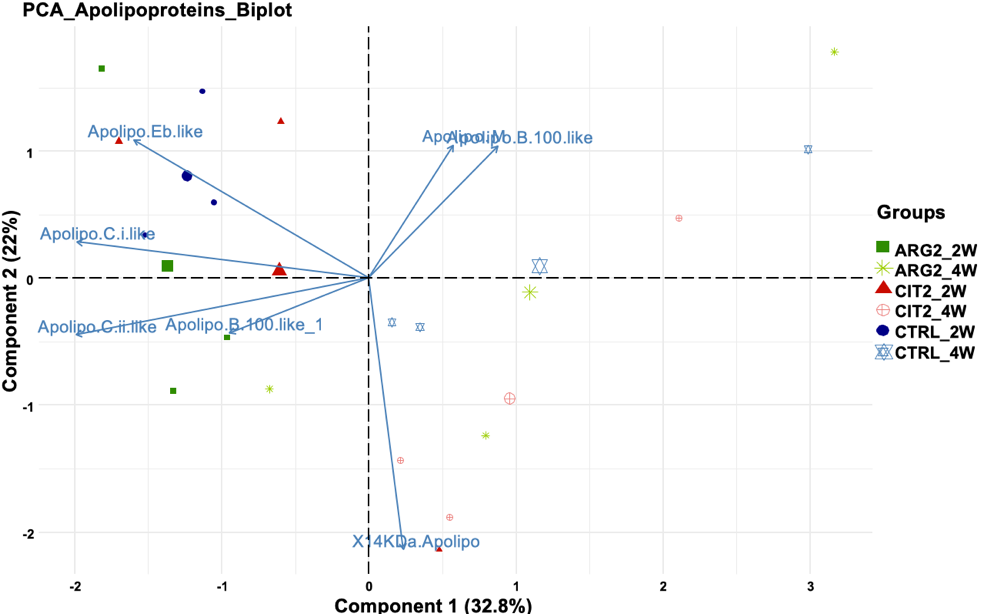


C


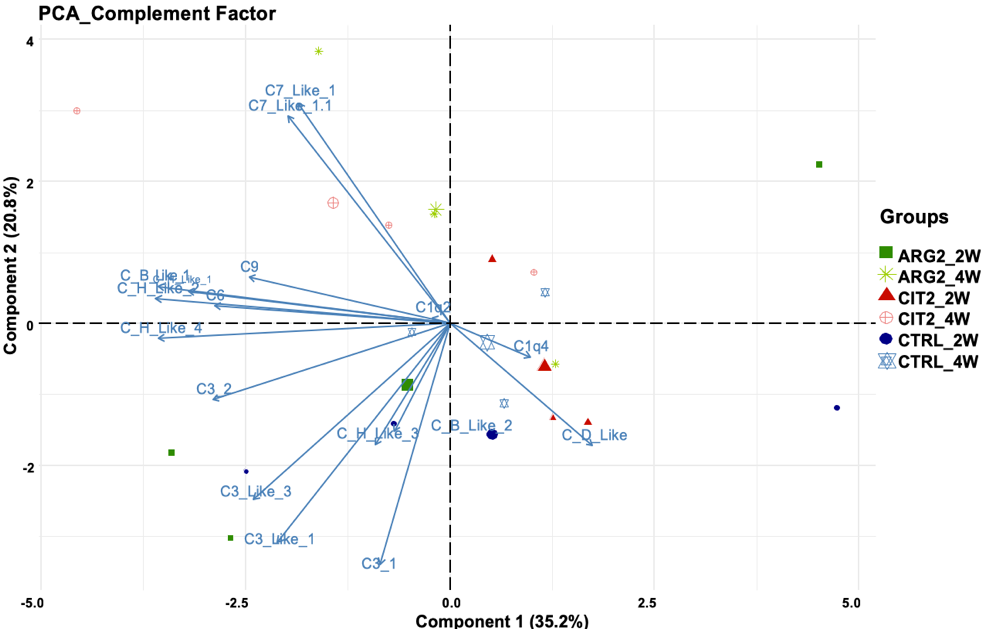


**FIGURE S1.** Principal component analysis (PCA) of plasma proteomics signatures of fishes fed the experimental diets. List of the 94 identified proteins can be found on table S3. **(A)** PCA score plots of all proteins analysed along the two main components, **(B)** PCA score plots of proteins belonging to the apolipoproteins family. **(C)** PCA score plots proteins belonging to the complement family.


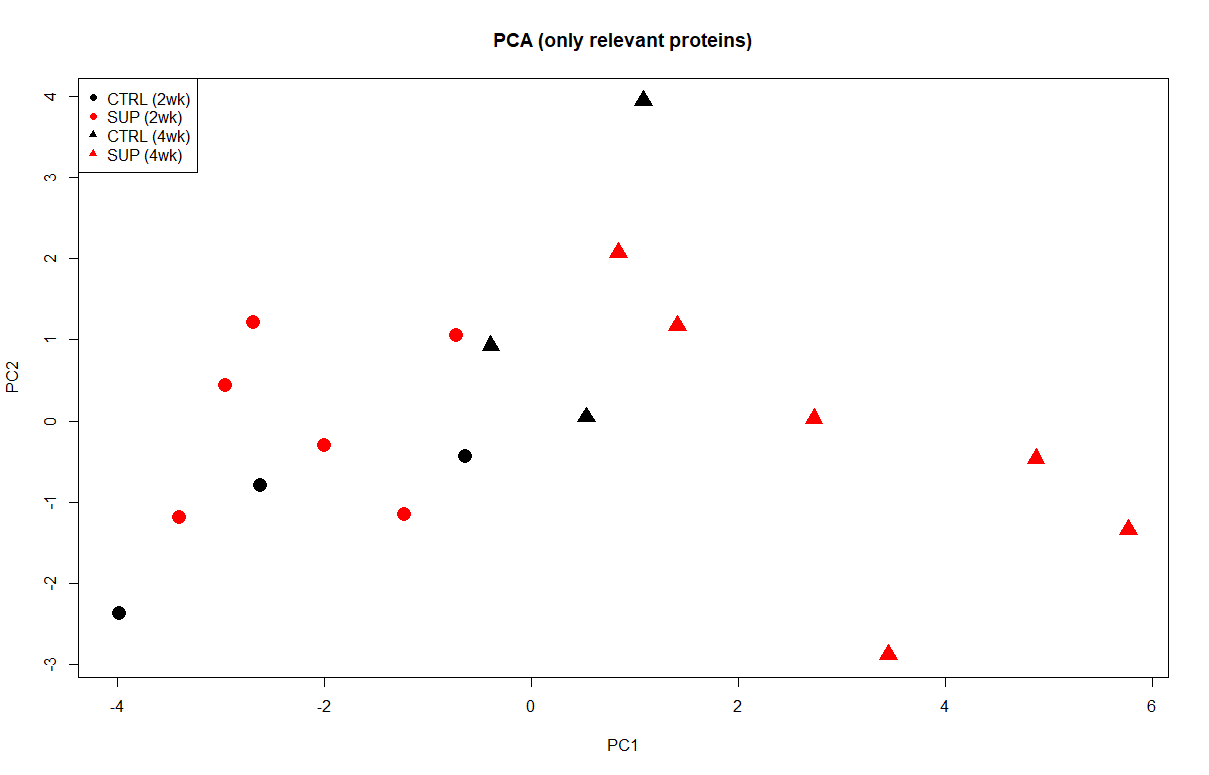


**FIGURE S2.** Principal component analysis (PCA) of plasma proteomic signatures of fish fed the experimental diets. PCA score plots of the 19 most relevant proteins analysed along the two main components, considering all supplemented diets as a pool designated as SUP.

**Table S1.** Proteins identified in plasma of fish fed all experimental diets.

| **Accession** | **Description** |
| --- | --- |
| Sa_26882.2.1 | complement C3-like |
| Sa_21997.2.1 | fibrinogen beta chain |
| Sa_9899.8.1 | ceruloplasmin-like |
| Sa_19340.12.5 | complement factor B-like |
| Sa_33122.4.1 | 14 kDa apolipoprotein |
| Sa_44333.2.1 | kininogen-1-like |
| Sa_19340.12.7 | complement factor B-like |
| Sa_27286.2.1 | beta-2-glycoprotein 1-like |
| Sa_26882.1.1 | complement component C3 |
| Sa_944.7.1 | N-acetylmuramoyl-L-alanine amidase-like |
| Sa_23416.2.2 | Angiotensinogen |
| Sa_32547.1.2 | apolipoprotein Eb-like |
| Sa_22208.2.1 | apolipoprotein B-100-like |
| Sa_1704.2.1 | complement factor H-like |
| Sa_23268.1.1 | complement component C6 |
| Sa_12582.3.1 | sex hormone-binding globulin |
| Sa_27317.1.1 | complement component C9 |
| Sa_48024.10.1 | alpha-1-acid glycoprotein 1-like |
| Sa_2627.1.1 | complement factor H-like |
| Sa_30666.1.1 | proteoglycan 4-like isoform X2 |
| Sa_27286.3.1 | beta-2-glycoprotein 1-like |
| Sa_1704.3.2 | complement factor H-like |
| Sa_16459.1.1 | lumican |
| Sa_37816.2.2 | cartilage acidic 1-like |
| Sa_32547.2.1 | apolipoprotein C-I-like |
| Sa_1704.1.1 | complement factor H-like |
| Sa_22432.2.1 | complement C3-like |
| Sa_8250.3.1 | serum amyloid P-component-like |
| Sa_48024.9.1 | alpha-1-acid glycoprotein 1-like |
| Sa_41650.2.1 | alpha-2-antiplasmin-like |
| Sa_27134.1.1 | plexin-A4 isoform X1 |
| Sa_13538.10.1 | inter-alpha-trypsin inhibitor heavy chain H2 |
| Sa_22432.3.1 | Complement C3 |
| Sa_736.1.1 | immunoglobulin light chain isotype partial |
| Sa_19875.1.1 | complement factor D-like |
| Sa_15521.3.1 | catechol O-methyltransferase domain-containing 1-like |
| Sa_41650.1.1 | pigment epithelium-derived factor |
| Sa_25136.3.1 | phosphoglycerate mutase 2 |
| Sa_16666.6.1 | coagulation factor XIII A chain |
| Sa_46975.2.1 | myelin zero 2 |
| Sa_22208.1.1 | apolipoprotein B-100-like |
| Sa_15740.1.1 | Cytosolic phospholipase A2 gamma |
| Sa_17989.1.1 | platelet glycoprotein V-like |
| Sa_33.3.1 | Interleukin-4 receptor subunit alpha |
| Sa_8915.3.1 | ependymin-1-like |
| Sa_33122.3.1 | apolipoprotein C-II-like |
| Sa_18092.7.1 | Z-dependent protease inhibitor-like |
| Sa_17271.1.1 | triosephosphate isomerase |
| Sa_16733.6.1 | apolipoprotein M |
| Sa_4480.1.2 | beta-enolase |
| Sa_4774.6.1 | peroxiredoxin 2 |
| Sa_23268.2.1 | complement component C7-like |
| Sa_34833.4.1 | cytoplasmic 2 |
| Sa_52479.3.1 | fibrinogen 1 |
| Sa_31172.5.1 | glyceraldehyde-3-phosphate dehydrogenase |
| Sa_9379.3.3 | glycogen muscle form |
| Sa_43047.2.1 | complement C1q 2 |
| Sa_7568.2.1 | alpha-2-HS-glyco -like |
| Sa_45700.1.1 | Ubiquitin-40S ribosomal S27a |
| Sa_46414.1.1 | complement component C7-like |
| Sa_4795.1.1 | phosphoglucomutase-1 |
| Sa_4196.3.1 | 3-hydroxyanthranilate 3 4-dioxygenase |
| Sa_10407.1.1 | plexin-A4 |
| Sa_14444.2.1 | histone H4- partial |
| Sa_18064.4.1 | Peptide-N(4)-(N-acetyl-beta-D-glucosaminyl)asparagine amidase F |
| Sa_35438.1.1 | glutathione peroxidase 3 |
| Sa_17527.8.1 | creatine kinase B-type isoform X1 |
| Sa_20361.1.1 | laminin subunit gamma-3-like |
| Sa_46557.1.1 | lysyl oxidase homolog 4-like |
| Sa_19603.2.1 | cadherin-5-like |
| Sa_4088.1.1 | histone H3 |
| Sa_20086.1.1 | Carboxypeptidase Z |
| Sa_19293.4.1 | coagulation factor VIII-like |
| Sa_13814.1.1 | intelectin-like |
| Sa_10005.8.1 | tubulin beta chain |
| Sa_24913.2.4 | coagulation factor IX |
| Sa_14443.1.1 | histone H2A-like |
| Sa_9500.7.1 | thrombospondin-4-B-like |
| Sa_11647.2.1 | vitronectin-like |
| Sa_8250.6.4 | RIIa domain-containing 1 isoform X4 |
| Sa_1942.4.1 | fucose mutarotase isoform X1 |
| Sa_18502.2.1 | leucine-rich PPR motif-containing mitochondrial |
| Sa_28742.5.1 | TBC1 domain family member 10A-like |
| Sa_40837.1.1 | hyaluronan and proteoglycan link 1-like |
| Sa_13080.2.1 | complement C1q 4 |
| Sa_27877.1.1 | threonine-protein kinase 494 |
| Sa_17242.1.2 | 14-3-3 epsilon isoform X1 |
| Sa_30036.2.1 | nidogen-1 |
| Sa_18106.7.1 | LETM1 and EF-hand domain-containing mitochondrial isoform X1 |
| Sa_5819.3.1 | 78 kDa glucose-regulated |
| Sa_12688.1.1 | cdc42-interacting 4 homolog isoform X2 |
| Sa_20976.2.1 | elongation factor 2-like |

**Table S2.** Quantitative expression of immune-related genes in the head-kidney of gilthead seabream fed the dietary treatments during 2 and 4 weeks.

| **2 weeks** | | | | | |  | **4 weeks** | | | | |
| --- | --- | --- | --- | --- | --- | --- | --- | --- | --- | --- | --- |
| Gene symbol | CTRL | ARG1 | ARG2 | CIT1 | CIT2 | CTRL | | ARG1 | ARG2 | CIT1 | CIT2 |
| *il-10* | 0.0003 ± 0.0001 | 0.0003 ± 0.0001**^#^** | 0.0004 ± 0.0002 | 0.0004 ± 0.0001 | 0.0003 ± 0.0002 | 0.0001 ± 0.0000**^ab^** | | 0.0001 ± 0.0000**^b*^** | 0.0000 ± 0.0000 **^ab^** | 0.0000 ± 0.0000 **^ab^** | 0.0000 ± 0.0000**^a^** |
| *il-34* | 0.0120 ± 0.0023 | 0.0098 ± 0.0026 | 0.0104 ± 0.0031 | 0.0144 ± 0.0057 | 0.0088 ± 0.0028 | 0.0166 ± 0.0060 | | 0.0121 ± 0.0051 | 0.0115 ± 0.0033 | 0.0139 ± 0.0037 | 0.0116 ± 0.0047 |
| *Il4-13* | 0.0021 ± 0.0012 | 0.0021 ± 0.0010 | 0.0023 ± 0.0007 | 0.0021 ± 0.0008 | 0.0018 ± 0.0010 | 0.0027 ± 0.0013 | | 0.0019 ± 0.0009 | 0.0016 ± 0.0003 | 0.0022 ± 0.0007 | 0.0018 ± 0.0011 |
| *il-1β* | 0.0034 ± 0.0036 | 0.0052 ± 0.0041 | 0.0069 ± 0.0055 | 0.0036 ± 0.0025 | 0.0072 ± 0.0062 | 0.0054 ± 0.0040 | | 0.0081 ± 0.0065 | 0.0069 ± 0.0087 | 0.0048 ± 0.0063 | 0.0011 ± 0.0011 |
| *cd8α* | 0.0004 ± 0.0002 | 0.0004 ± 0.0002 | 0.0004 ± 0.0002 | 0.0004 ± 0.0002 | 0.0004 ± 0.0002 | 0.0003 ± 0.0002 | | 0.0002 ± 0.0002 | 0.0002 ± 0.0001 | 0.0002 ± 0.0003 | 0.0001 ± 0.0001 |
| *cd4* | 0.0805 ± 0.0294 | 0.0717 ± 0.0243 | 0.0683 ± 0.0278 | 0.0647 ± 0.0320 | 0.0441 ± 0.0096 | 0.0719 ± 0.0186 | | 0.0912 ± 0.0317 | 0.0754 ± 0.0192 | 0.0796 ± 0.0395 | 0.0669 ± 0.0438 |
| *tnf-α* | 0.0001 ± 0.0001 | 0.0002 ± 0.0001 | 0.0002 ± 0.0001 | 0.0002 ± 0.0001 | 0.0002 ± 0.0001 | 0.0001 ± 0.0000 | | 0.0001 ± 0.0001 | 0.0001 ± 0.0001 | 0.0001 ± 0.0001 | 0.0001 ± 0.0001 |
| *IgM* | 1.1329 ± 0.5657 | 1.6613 ± 0.5610 | 1.1371 ± 0.4185 | 1.3900 ± 0.6531 | 1.2833 ± 0.4807 | 1.4978 ± 0.8463 | | 1.4978 ± 0.8463 | 1.4978 ± 0.8463 | 1.4978 ± 0.8463 | 1.4978 ± 0.8463 |
| *tcr* | 0.1041 ± 0.0403 | 0.1012 ± 0.0255 | 0.0855 ± 0.0290 | 0.0987 ± 0.0531 | 0.0639 ± 0.0221 | 0.0951 ± 0.0665 | | 0.1011 ± 0.0632 | 0.1176 ± 0.0425 | 0.1032 ± 0.0782 | 0.0861 ± 0.0645 |
| *csfr* | 0.0002 ± 0.0002 | 0.0002 ± 0.0001 | 0.0002 ± 0.0000 | 0.0002 ± 0.0001 | 0.0002 ± 0.0001 | 0.0002 ± 0.0002 | | 0.0003 ± 0.0002 | 0.0003 ± 0.0002 | 0.0004 ± 0.0002 | 0.0002 ± 0.0001 |
| *tgfb* | 0.0412 ± 0.0106 | 0.0447 ± 0.0106 | 0.0424 ± 0.0089 | 0.0451 ± 0.0094 | 0.0369 ± 0.0087 | 0.0366 ± 0.0216 | | 0.0418 ± 0.0193 | 0.0489 ± 0.0227 | 0.0421 ± 0.0182 | 0.0407 ± 0.0270 |
| *arg-II* | 0.0170 ± 0.0079 | 0.0179 ± 0.0029 | 0.0203 ± 0.0080 | 0.0236 ± 0.0108 | 0.0161 ± 0.0068 | 0.0201 ± 0.0092 | | 0.0202 ± 0.0069 | 0.0202 ± 0.0080 | 0.0267 ± 0.0351 | 0.0115 ± 0.0039 |

Values are presented as means ± SD (*n* = 9). *P*-values from two-way ANOVA (*p* ≤ 0.05). Tukey *post-hoc* test was used to identify differences in the experimental treatments. Different lowercase letters stand for significant differences among dietary treatments for the same time. Different capital letters indicate differences among diets regardless time or time regardless diets. Different symbols indicate difference among time for the same dietary treatment.

| Two-way ANOVA | | | | | | | | | | | | |
| --- | --- | --- | --- | --- | --- | --- | --- | --- | --- | --- | --- | --- |
| Gene symbol |  |  |  |  | Time | |  | Diet | | | | |
|  | **Time** | **Diet** | **Time x diet** |  | **2 weeks** | **4 weeks** |  | **CTRL** | **ARG1** | **ARG2** | **CIT1** | **CIT2** |
| *il-10* | 0.005 | 0.037 | 0.021 |  | - | - |  | - | - | - | - | - |
| *il-34* | 0.039 | 0.021 | 0.577 |  | - | - |  | AB | AB | AB | B | A |
| *Il4-13* | 0.867 | 0.425 | 0.445 |  | - | - |  | - | - | - | - | - |
| *il-1β* | 0.997 | 0.392 | 0.101 |  | - | - |  | - | - | - | - | - |
| *cd8α* | 0.004 | 0.163 | 0.738 |  | B | A |  | - | - | - | - | - |
| *cd4* | 0.105 | 0.161 | 0.635 |  | - | - |  | - | - | - | - | - |
| *tnf-α* | <0.001 | 0.544 | 0.755 |  | B | A |  | - | - | - | - | - |
| *IgM* | 0.245 | 0.791 | 0.088 |  | - | - |  | - | - | - | - | - |
| *tcr* | 0.384 | 0.506 | 0.786 |  | - | - |  | - | - | - | - | - |
| *csfr* | 0.003 | 0.306 | 0.284 |  | A | B |  | - | - | - | - | - |
| *tgfb* | 0.991 | 0.721 | 0.851 |  | - | - |  | - | - | - | - | - |
| *arg-II* | 0.815 | 0.234 | 0.908 |  | - | - |  | - | - | - | - | - |
